# Middle cerebral artery dissection: pathophysiology, diagnosis, and therapeutic options

**DOI:** 10.3389/fneur.2025.1622630

**Published:** 2025-08-01

**Authors:** Xiangpeng Yao, Niao Yang, Nao Yan

**Affiliations:** ^1^The Second Clinical College of Wuhan University, Wuhan, Hubei, China; ^2^Department of Cardiology, Wuhan University of Science & Technology, Hanyang Hospital, Wuhan, Hubei, China; ^3^Department of Neurology, Zhongnan Hospital of Wuhan University, Wuhan, Hubei, China

**Keywords:** middle cerebral artery dissection, risk factors, pathophysiology, imaging characteristics, treatment

## Abstract

Middle cerebral artery dissection (MCAD) is a rare cerebrovascular disorder that can lead to ischemic stroke or hemorrhagic events. Although its exact incidence remains unclear, the detection rate of MCAD has increased in recent years with advancements in imaging technology. Based on a systematic literature review of MEDLINE/PubMed, the Cochrane Library, Web of Science, and EMBASE, combined with the latest guidelines on intracranial artery diseases and stroke management, this article comprehensively reviews the pathophysiological mechanisms, imaging characteristics, and treatment strategies for MCAD.

## Introduction

Middle cerebral artery (MCA) dissection is an exceedingly rare clinical vascular disorder, predominantly affecting younger individuals. Both ischemic and hemorrhagic manifestations may occur, contributing to its diagnostic and therapeutic complexity. With advancements in diagnostic and therapeutic techniques, clinical management of middle cerebral artery dissection (MCAD) has achieved modest progress. However, due to the scarcity of reported cases and limited collective experience, standardized diagnostic criteria and comprehensive treatment guidelines for MCAD remain to be established.

In clinical practice, the diagnosis of MCAD is primarily established through a comprehensive assessment incorporating characteristic clinical manifestations, detailed patient history, and distinctive neuroimaging findings. The treatment of middle cerebral artery dissection is currently extrapolated from established protocols for intracranial artery dissection and extracranial cervical artery dissection, primarily involving medical therapy and endovascular intervention. This review focuses on systematically summarizing: (1) Pathophysiology, (2) diagnostic modalities and characteristic clinical presentations, and (3) current therapeutic strategies for MCAD.

## Anatomy of the middle cerebral artery

The middle cerebral artery is the largest and most complex branch of the internal carotid artery (ICA), serving as the predominant vascular supply to critical regions of the cerebrum, including: (1) the lateral frontal, parietal, and temporal lobes; (2) the basal ganglia via its lenticulostriate perforators; and (3) key functional areas such as the motor cortex, Broca’s area, and Wernicke’s area. It is a favored site for a number of common diseases such asthromboembolic stroke (accounting for ~40% of all ischemic strokes), saccular aneurysms (most frequently occurring at the M1-M2 bifurcation), arteriovenous malformations (particularly those involving the peri-Sylvian region), and tumor-related vascular encasement (notably in glioblastoma and metastatic lesions). From an evolutionary perspective, the middle cerebral artery is the blood supply to the expanding cerebral cortex. And it is a branch of both the ACA. At the embryonic stage, the MCA emanates from the ICA and gradually fuses from a tuft into a single tubular structure (1–3).

Despite the anatomical proximity of frontoparietal and temporal cortical branches, critical microvascular observations reveal two fundamental characteristics: (1) no functional anastomoses exist between adjacent cortical vessels, creating discrete watershed zones at territorial boundaries, and (2) complete vascular segregation occurs both between the superior (frontoparietal) and inferior (temporal) trunk systems, and among individual perforating lenticulostriate branches. This dual isolation results in strictly compartmentalized perfusion territories that explain the characteristic infarct patterns observed in clinical practice.

The middle cerebral artery is divided into four segments:

The M1 segment (pterygoid/horizontal segment) originates from the internal carotid artery (ICA) bifurcation and courses laterally within the Sylvian fissure, maintaining a strictly horizontal trajectory without crossing vessels. This segment gives rise to two critical branches: (1) medial lenticulostriate perforators (‘bean arteries’) supplying the basal ganglia, and (2) distal cortical branches including the orbitofrontal artery which perfuses the lateral orbital gyrus and inferolateral frontal lobe. Anatomical variations exist, with ~15–20% of cases demonstrating trifurcation into distinct frontal, parietal, and temporal trunks. The M1 designation persists until bifurcation/trifurcation, with significant interindividual length variability (range: 12–22 mm). Notably, the Sylvian fissure remains devoid of traversing arterial anastomoses, enforcing strict vascular territorial boundaries.

As the MCA’s first post-bifurcation segment, the M2 demonstrates three surgically critical features: (1) its asymmetric, U-shaped insular course creates predictable anatomical relationships for microsurgical navigation, (2) the anterior temporal artery origin marks a key landmark for temporal lobe vascularization, and (3) its 6–8 terminal branches establish non-overlapping perfusion zones for the insular cortex and opercula, explaining the precise neurological deficits observed in selective occlusions.

The M3 segment (operculum segment) extends from the circular sulcus of the insula to the cortical surface of the Sylvian fissure, comprising ascending cortical branches that originate from the M2 division. These branches include: (1) the orbitofrontal artery, supplying the inferior frontal gyrus and pars orbitalis; and (2) the frontoparietal ascending arteries, which further divide into the precentral, central, and postcentral arteries. These subdivisions precisely vascularize the precentral gyrus (motor cortex), central sulcus (sensorimotor junction), and postcentral gyrus (primary somatosensory cortex), respectively. The M3 arteries demonstrate a characteristic ‘candelabra’ branching pattern as they emerge from the Sylvian fissure, with minimal anastomoses between adjacent vessels, resulting in well-defined functional territories.

The M4 segment represents the terminal cortical distribution of the middle cerebral artery (MCA), emerging from the Sylvian fissure to supply the lateral hemispheric surface. Key branches include: (1) the angular artery, which courses posteriorly to vascularize the angular gyrus (Brodmann area 39); (2) the posterior parietal artery, perfusing the superior parietal lobule and intraparietal sulcus; and (3) the posterior temporal artery, supplying the middle and inferior temporal gyri. These branches exhibit minimal anastomoses with adjacent vascular territories, creating sharply demarcated watershed zones at the parietal-occipital and temporal-occipital junctions. The M4 vessels demonstrate a characteristic ‘fan-shaped’ radiation over the cortical convexity, with branching patterns that correlate strongly with gyral anatomy (1, 4–6).

**Figure 1:**
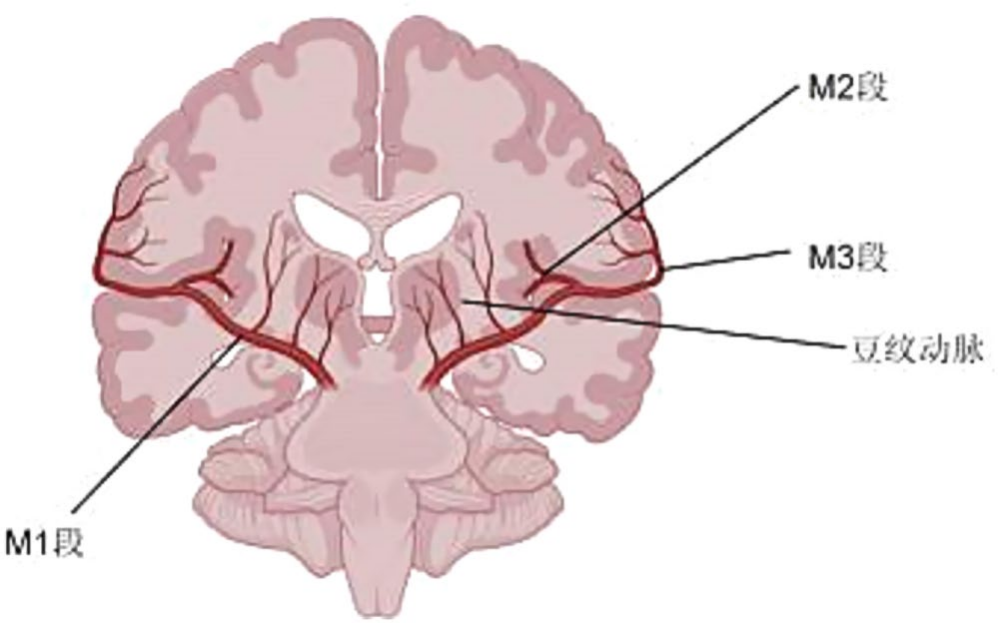
Segmentation of the middle cerebral artery.

MCA has a few anomalies on it. An accessory MCA is a variant that arises only from the ACA, and any MCA variant that arises from the ICA should be classified as a duplicate MCA. Originating from a single trunk is the MCA fenestration, which has a fenestration, while the swig-like MCA has no trunk and is replaced by a twig-like arterial network (Figure 1) (5).

### Risk factors

MCAD shares overlapping risk factors with cervical artery dissection (CAD) and intracranial artery dissection (ICAD), which can be categorized as follows: age and sex, environmental triggers (best documented), genetic predisposition (hypothesis-driven), underlying arteriopathy (pathology-supported) (7, 8).

#### Age and sex

The incidence of CeAD is predominantly in young and middle-aged people, with the average age of onset of the disease being 45 years, and in surveys in which hospital patients were the subject of the study, males predominated (7).

Environmental Triggers (best documented).

Migraine is an important warning sign of CeAD and is a common environmental factor. A history of migraine, especially migraine without aura, is more common in patients with CeAD than in patients with ischaemic stroke without CeAD. Cervical spine injury is a factor that should not be overlooked, although 60 % of CeAD is spontaneous. Close to half of CeAD patients have a history of a mechanically triggered event. (e.g., minor cervical spine motion injuries, severe penetrating injuries), with cervical spine manipulation and sports-type traumas being the most prevalent (7, 8). There are many other injuries: childbirth, coughing, sneezing, vomiting and motor vehicle accidents, which can cause major trauma. There are many other injuries: childbirth, coughing, sneezing, vomiting and motor vehicle accidents, which can cause major trauma. Infections have been reported to be more common in patients with AeCD, suggesting that the inflammatory state may be a trigger. The mechanisms behind this may be endothelial damage and prothrombotic is worth noting that typical pro-atherosclerotic risk factors have a neutral or inverse relationship with CeAD. One of the surprises is hypertension, which is viewed as a major susceptibility factor for CeAD. The reason for this may be that elevated blood pressure increases the risk of disease by increasing the stiffness of the carotid arteries. At the same time, hypercholesterolaemia, obesity and overweight are at lower risk. In addition, seasonal factors can affect the incidence of CeAD, with autumn and winter being more likely. The mechanism may be related to blood pressure and infection (8, 9).

#### Genetic predisposition (hypothesis-driven)

Genetic contributions to cervical artery dissection (CeAD) operate through two principal mechanisms: (1) monogenic disorders conferring high individual risk, and (2) oligogenic/multifactorial predisposition in familial cases. The prototypical monogenic form is vascular Ehlers-Danlos syndrome (vEDS), where COL3A1 mutations cause type III collagen deficiency, resulting in fragile arterial walls and a 15–20% lifetime CeAD risk, typically accompanied by red flags such as familial arterial rupture, characteristic facies (thin nose/lips), and translucent skin. Similarly, Marfan syndrome (FBN1 mutations) demonstrates 5–8% CeAD prevalence via elastic fiber fragmentation, often with concurrent aortic root dilation or ectopia lentis. In contrast, disseminated familial CeAD (1–3% of cases) exhibits incomplete penetrance (40–60%) through combined rare variants (COL5A1/COL5A2) and common SNPs (PHACTR1, HTRA1), where environmental triggers (minor trauma, postpartum states) interact with genetic susceptibility to precipitate dissection. This genetic architecture underscores the need for tailored screening—COL3A1/FBN1 testing in syndromic cases versus polygenic risk assessment in multifactorial families (10, 11).

#### Underlying arteriopathy (pathology-supported)

While overt collagen vascular disorders (e.g., Ehlers-Danlos or Marfan syndromes) are identified in only 1–5% of cervical artery dissection (CeAD) cases, subtle connective tissue abnormalities—including joint hypermobility, easy bruising, and poor wound healing—are observed in 50–96% of spontaneous CeAD patients. These findings, coupled with frequent vascular tortuosity and aortic root dilation, suggest most ‘spontaneous’ CeAD occurs in the context of systemic but subclinical connective tissue dysfunction. This predisposition converges with fibromuscular dysplasia (FMD), a non-inflammatory arteriopathy demonstrating bidirectional associations with CeAD: 10% of FMD patients develop CeAD (predominantly carotid arteries), while advanced imaging reveals FMD in ~40% of spontaneous CeAD cases (8% in the IPSYS CeAD cohort). Clinically, FMD-associated CeAD exhibits distinct features: (1) higher rates of symptomatic vascular events and endovascular interventions, (2) comorbid migraines and intracranial aneurysms, and (3) reduced association with traumatic triggers compared to non-FMD CeAD. Collectively, these observations position CeAD along a spectrum of underlying vasculopathies ranging from mild connective tissue fragility to defined arteriopathies like FMD (10, 12–14).

Few studies have examined the risk factors for both intracranial and carotid artery dissection, and from the few studies that have done so, it has been concluded that there is no difference in the distribution of vascular risk factors between the two groups, with the exception of one study that showed a higher prevalence of hypertension in patients with intracranial artery dissection. However, in this study, patients with intracranial artery dissection were older than control participants (mean 48 years vs. 37 years).

Similar to the risk factors of carotid artery dissection, the risk factors of intracranial artery dissection can also be divided into these three parts, the specific content and mechanism of which have a lot of similarities with the former, and will not be expanded in detail here, but there are some differences. For example, the genetic factors of intracranial artery dissection have not been investigated so far (Table 1) (15).

**Table 1:** Risk factors for MCAD.

Categories of risk factors	Specific factors	Possible mechanism/pathophysiology	Related clinical manifestations	Intervenable	Diagnostic relevance	Support evidence levels	Reference
Immutable factor	Age (45 years)	Vessel wall mesostructure more vulnerable in young patients	Spontaneous dissection	It cannot be intervened	high	Middle-aged and older	(7)
Immutable factor	Gender (slightly more men)	May be associated with hormone levels or risk of trauma	Traumatic/non-traumatic dissection	It cannot be intervened	low	middle	(7)
hereditary factor	Hereditary connective tissue	Structural abnormalities of the vessel wall (e.g., Ehlers-Danlos syndrome, Marfan syndrome)	Recurrent arterial dissection	Some intervention	high	high	(10, 11)
Vascular-related factors	Hypertension	Increased haemodynamic stress and vessel wall damage	The interlayer progresses or ruptures	It can be intervened	middle	high	(9)
Vascular-related factors	Inflammatory vascular diseases (e.g., SLE, vasculitis)	Inflammation destroys the integrity of the vessel wall	Multiple vascular lesions	Some intervention	low	middle	(9)
environmental factor	Head and neck trauma	Tearing of blood vessel wall due to direct external force	Post-traumatic compartment syndrome	Some intervention	high	high	(10)
Environmental factor	Cervical massage/strenuous exercise	Vascular traction injury from excessive neck rotation or stretching	Spontaneous dissection	Avoidable	middle	middle	(50)
Potential arterial lesion	Abnormal blood flow shear	Turbulence or high flow rate causes damage to the inner lining of blood vessels	Stratified expansion or thrombosis	Some intervention	low	middle	(51)
environmental factor	Migraine (especially migraine with aura)	Vasospasm or endothelial dysfunction	Non-traumatic dissection	Some intervention	middle	middle	(7, 8)
environmental factor	Infections (e.g., COVID-19)	Vasculitis or endothelitis	Vascular complications after infection	It can be intervened	low	Low to medium	(8)
Potential arterial lesion	Oral contraceptives/hormone replacement therapy	May affect vascular wall structure or coagulation function	Spontaneous dissection in young women	It can be intervened	low	middle	(21)

#### Nosogenesis

**Figure 2:**
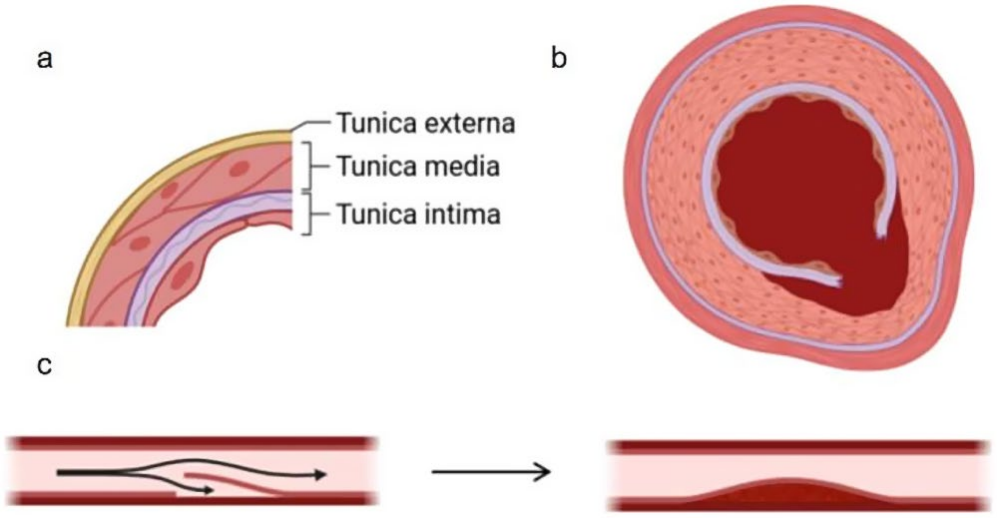
**(a)** Shows the stratification of the middle artery in the brain, and **(b)** shows the cross section of the false cavity formed after the rupture of the inner membrane and the blood enters. **(c)** Shows the formation of the false cavity and the stenosis of the lumen.

The MCA wall exhibits a layered architecture that dictates its functional and pathological behavior. Beginning at the lumen, the *tunica intima* (endothelium) comprises a single cell layer anchored to a thin basement membrane, critical for maintaining blood–brain barrier integrity and regulating vascular tone through nitric oxide secretion—its focal denudation often initiates dissection. Adjacent to this, the *internal elastic lamina (IEL)* forms a 5–8 μm fenestrated elastin sheet; rupture of this layer defines classical arterial dissection (Type 1), while its thinning predisposes to aneurysm formation. The middle layer, *tunica media*, consists of 15–20 smooth muscle cell layers embedded in a type III collagen matrix (notably vulnerable in Ehlers-Danlos syndrome). Structurally, the MCA’s media is thinner (0.1–0.3 mm) than extracranial arteries and lacks vasa vasorum, rendering it dependent on luminal perfusion. Unlike extracranial vessels, intracranial arteries (MCA, ACA, PCA) lack an external elastic lamina (EEL), which explains their heightened susceptibility to dissection propagation. Outermost, the *tunica adventitia*—composed of type I collagen and autonomic nerve endings—serves to contain intramural hemorrhage in dissections and becomes infiltrated in vasculitic processes. Pathogenesis of arterial dissection is initiated by structural failure at the intimal-medial junction, characterized by rupture of the internal elastic lamina and degradation of the tunica media’s extracellular matrix. This breach permits extravasation of luminal blood into the vessel wall, forming an intramural hematoma that propagates along natural cleavage planes. The absence of external elastic lamina in intracranial arteries facilitates uncontrolled hematoma expansion, resulting in progressive separation of wall layers and creation of a false lumen. Continued hemorrhage is driven by both recurrent entry from the true lumen and secondary bleeding from vasa vasorum disruption. THis dynamic process culminates in either: (1) thromboembolism from false lumen thrombus formation, (2) critical stenosis due to true lumen compression, or (3) catastrophic vessel rupture upon adventitial penetration. The middle cerebral artery’s unique microanatomy—particularly its thin medial layer (0.1–0.3 mm) and lack of vasa vasorum—exacerbates these pathological cascades, explaining its disproportionate representation in intracranial dissections (Figure 2) (15–17).

#### Clinical symptom

Middle cerebral artery dissection represents a rare cerebrovascular entity, exhibiting distinct clinicoradiological patterns compared to posterior circulation dissections. Current evidence demonstrates a strong anatomical predilection for ischemic manifestations in anterior circulation dissections, while posterior intracranial dissections (vertebrobasilar system) predominantly manifest with subarachnoid hemorrhage. This dichotomy reflects fundamental differences in vessel wall architecture—notably the absence of external elastic lamina and thinner medial layers in posterior circulation arteries, increasing their susceptibility to hemorrhagic transformation. With aneurysmal dilatation typically manifest as hemorrhagic phenomena due to rupture of the weakened vessel wall, whereas non-aneurysmal middle cerebral artery (MCA) dissections predominantly present with ischemic symptoms secondary to thromboembolism or flow-limiting stenosis. Cerebral artery (MCA) dissection can precipitate ischemic stroke through severe luminal stenosis or complete vascular occlusion. However, subarachnoid hemorrhage (SAH) may rarely occur due to the unique histopathological vulnerability of intracranial arteries—specifically the MCA’s thin medial layer (0.1–0.3 mm) and absence of external elastic lamina. This structural deficiency predisposes to vessel wall rupture under hemodynamic stress. Lin et al.’s seminal case series of 23 isolated MCA dissections demonstrated a marked clinical dichotomy, with hemorrhagic presentation (subarachnoid hemorrhage) occurring in 12% of cases versus ischemic manifestations in 88%. This distribution reflects two distinct underlying pathomechanisms: (1) thromboembolism originating from the dissection flap or intramural hematoma, accounting for the ischemic majority, and (2) adventitial rupture leading to hemorrhage in the minority of cases. Middle cerebral artery dissection-induced cerebral infarction typically manifests with characteristic neurological deficits including contralateral hemiparesis, ipsilateral central facial palsy, and varying degrees of consciousness impairment (18, 19).

In some patients with cerebral artery dissection, headache can be the only symptom. The location is often confined to the side of the affected artery, with almost no bilateral pain. The onset of pain is sudden and intense, described as lightning-like. The severity of the pain is closely related to posture; when the patient lies supine, the pain eases. When the patient lies on the side opposite to the dissection, the pain worsens, typically manifesting as pulsating or tight pain in the ipsilateral parietal and temporal lobes. When the patient lies on the opposite side of the dissection, the pain may disappear. The direct cause and mechanism of the pain remain unclear, but there are currently three main theories: 1. When lying on the same side as the dissection, gravitational forces increase the arterial perfusion pressure, causing temporary dilation of the vessel and exacerbating the pain; 2. Pain is caused by direct tearing of the vessel wall; 3. Inflammatory neurotransmitters are released from nerve endings around the vessel wall. Other symptoms include seizures, nausea/vomiting, tinnitus, and no neurological deficits associated with stroke (20).

Typical symptoms of middle cerebral artery dissection include:

Sudden focal neurological deficit: manifested by hemiparesis (face and upper limbs more than lower limbs), hemiplegia or isotropic hemianopsia on the side contralateral to the lesion.

Acute headache: mostly located in the temporal or periorbital region on the side of the lesion, with tearing or throbbing pain;

Language disorders: e.g. aphasia (Broca’s aphasia, Wernicke’s aphasia) or dysarthria;

Seizures: present as partial seizures or generalized tonic–clonic seizures;

Impaired consciousness: e.g. drowsiness, coma;

Hemorrhagic manifestations: e.g. sudden severe headache, vomiting, cervical rigidity, suggestive of subarachnoid hemorrhage.

#### Imaging features

In the extensive literature we reviewed, middle cerebral artery dissection in the brain is most commonly found in the M1 and M2 segments. Imaging and other examination methods clearly indicate a high likelihood of MCA dissection. Direct signs of MCAD include: intimal flap, double lumen sign, and intraluminal hematoma; indirect signs include beaded appearance or segmental stenosis, pseudoaneurysm, and vascular occlusion. The following describes these examinations and their corresponding characteristics.

#### Intimal flap

Definition: A tear in the tunica intima of the arterial wall, creating a mobile flap that separates the true lumen from the false lumen.

Imaging: Visible as a thin, linear filling defect on DSA (Digital Subtraction Angiography), CTA, or MRI.

#### Double lumen sign

Definition: Pathognomonic feature of dissection showing two distinct flow channels—true lumen (narrowed by compression) and false lumen (thrombus or slow flow).

Imaging: Best seen on DSA or high-resolution MRI (T1 fat-sat with intramural hematoma).

#### Intramural hematoma

Definition: Hemorrhage within the arterial wall (media layer) due to rupture of the vasa vasorum or intimal tear.

Imaging: Crescent-shaped hyperintensity on T1-weighted MRI (methemoglobin signal).

#### String-of-beads sign

Definition: Alternating areas of stenosis and dilation due to segmental wall injury, commonly seen in fibromuscular dysplasia (FMD) or dissections.

Imaging: Appears on CTA/MRA/DSA as a beaded vascular contour.

#### Segmental stenosis

Definition: Focal or multifocal narrowing of the artery due to intimal thickening, hematoma compression, or vasospasm.

Imaging: Irregular lumen reduction on angiography (DSA/CTA/MRA).

#### Pseudoaneurysm

Definition: A rupture of all three arterial layers (intima, media, adventitia), contained by surrounding tissue, forming a sac-like outpouching.

Imaging: Contrast-filled sac without a true endothelial lining on DSA/CTA.

#### Vessel occlusion

Definition: Complete obstruction of the arterial lumen due to thrombosis, dissection progression, or embolus.

Imaging: Abrupt cutoff on angiography, with absent distal flow.

#### Arterial Wall thickening

Definition: Increased wall thickness from intramural hematoma, inflammation (vasculitis), or atherosclerosis.

Imaging: T1 MRI (hyperintense hematoma) or high-resolution vessel wall MRI (circumferential thickening).

#### Angiography

The gold standard for MCAD is DSA. DSA has the advantage of rapid imaging and can provide clinicians with dynamic vascular conditions throughout the process, and is currently considered the best method for clinical diagnosis of IAD (21).

It is a diagnostic tool when other imaging results are uncertain and is suitable for follow-up after endovascular treatment of suspected acute MCA dissection with ischemic stroke. Overall, the typical DSA presentation of MCA lesions is similar to that of extracranial artery dissection (22).

In patients with acute stroke secondary to middle cerebral artery dissection (MCAD), initial digital subtraction angiography (DSA) typically demonstrates two characteristic findings: (1) a distinct intimal flap, representing the separated inner vascular layer appearing as a linear filling defect, and (2) double lumen sign, the diagnostic gold standard observed in 50–60% of cases. The double lumen manifests as two parallel contrast-filled channels—a narrower true lumen with rapid antegrade flow and a wider false lumen showing delayed opacification due to stagnant blood flow. In complete vascular occlusion scenarios, DSA reveals abrupt contrast termination preceded by a focal intimal flap, indicating the dissection point. The intimal flap, a pathognomonic structural component formed by torn arterial intima, appears as a thin linear or curvilinear radiolucent band separating the lumina. Are also indirect signs, such as tapered stenosis demonstrating gradual vascular narrowing terminating in complete occlusion and the string-of-beads sign (observed in 60–70% of MCAD cases), characterized by alternating segments of irregular stenosis with adjacent proximal or distal dilatation. Pseudoaneurysm on DSA is characterized by contrast agent leakage and wide neck of the tumor. Stenosis of blood vessels is common in DSA, with the lesion site showing contrast agent filling defects, a shorter diameter than proximal and distal segments, and an irregular shape. Three-dimensional angiography and magnified angiography can also display similar results. As the disease progresses, the stenosis on DSA may worsen with the development of false aneurysms and irregularly wrapped flaps. Summary, if typical angiographic imaging signs such as the double lumen sign, string sign, bead sign, stenotic occlusive disease, or pseudoaneurysm are present, MCA dissection should be suspected. Among these, the double lumen sign and intimal flap are direct indicators of arterial dissection. If both signs are clearly demonstrated on DSA, MCAD can be diagnosed independently. If other indirect signs appear, other vascular diseases must be ruled out, and multiple examinations should be combined for diagnosis (18).

The advantage of DSA diagnosis MCAD is that it has the highest spatial distribution rate and can be treated synchronously. However, DSA also has its drawbacks; it is expensive and invasive, inevitably damaging the body and potentially causing complications. Additionally, it cannot assess the condition of the vessel wall, including intraluminal hematomas and thrombi, as it may misdiagnose MCAD when used in atypical presentations or unconventional sites. Angiography and MRA were compared with DSA to detect evidence of intraluminal hematoma and other lesions. Computed tomography (CT) angiography can show intimal tear the starting point of the dissection and thrombosis (low density filling defect) (21, 23, 24).

The advent of 4D-DSA technology has significantly enhanced the hemodynamic assessment of arterial dissections by providing time-resolved, three-dimensional angiographic data. As demonstrated by Chen et al. (2020), this advanced imaging modality offers superior discrimination between true and false lumina through dynamic flow characterization—specifically by demonstrating: (1) differential contrast filling kinetics (true lumen typically exhibits rapid antegrade flow, while false lumen shows delayed opacification with stasis), and (2) distinct temporal–spatial flow patterns during the cardiac cycle. These hemodynamic insights prove particularly valuable for therapeutic planning, as they allow for: (a) precise identification of entry/re-entry tears, (b) assessment of flow competition between lumina, and (c) evaluation of distal perfusion compromise. The technology’s clinical utility is underscored by its ability to modify treatment strategies in approximately 30–40% of complex dissection cases where conventional DSA findings remain ambiguous (25, 26).

#### Magnetic resonance imaging

Magnetic resonance imaging is a valuable technique for the diagnosis of intracranial arterial dissection. MRI or MRA is the preferred screening method for MCAD and is suitable for follow-up during acute and subacute phases as well as for assessment of lesions in the vessel wall. A direct sign of MCAD is an intracystic hematoma (27, 28). T1-weighted fat suppression signals are more likely to show intracystic hematomas. In the acute phase (less than 7 days after onset), it appears as T1WI iso/hypointensity, T2WI low signal in the subacute phase (1 to 4 weeks after onset), it shows T1WI high signal (due to methemoglobin formation), T2WI heterogeneous signal. In the chronic phase (more than 4 weeks after onset), T1/T2 signals decrease, with fibrotic changes. High signal of the interlayer aneurysm on T1WI indicates thrombosis, and TOF-MRA can show the tumor (27, 29).

The 7T3Dt1 spatial MRI shows an intracystic hematoma and residual lumen in the corresponding segment when MCA is stripped. A low-density circular structure is present within an eccentrically high-density ring, where the high-density signal indicates an eccentric wall hematoma, and the low-density signal suggests a residual lumen. The 3 T 3D T1-SPACE MRI can display intraluminal thrombus formation, appearing as a high-density circular shadow, while the low-density shadow is not prominent, meaning the residual lumen is unclear. This suggests that 7TMRI may have an advantage over 3TMRI in diagnosing MCAD, as it can show more pathological details (19).

HRMRI is a key technique for diagnosing MCAD, particularly in demonstrating the structure of blood vessel walls. By using black blood technology to suppress blood flow signals, it can clearly show direct signs such as hematomas within the vessel wall, intimal flaps, and double lumen signs. A crescent-shaped or arc-shaped low-density shadow appearing within the vessel wall is called the crescent sign. Medium signal patchy structures within the MCA are known as the flap sign. Compared to DSA, enhanced MRI scans show better enhancement of the intimal flap, providing a more accurate representation. MRI can directly observe intraluminal hematomas, which appear as thickened arterial walls with smooth edges. Intraluminal hematomas typically cause dilation of the outer lumen, with signal intensity varying over time. In the subacute phase, early in the chronic phase, they appear as high signals, often crescent-shaped, serving as direct evidence for diagnosing MCAD. On T1-weighted images, the two cavities separated by medium signal intimal plates are referred to as the double lumen sign. The true lumen is usually narrow and round, with higher blood flow velocity, appearing as high signals on MRI black blood sequences. The false lumen is wider and often crescent-shaped, resulting from intimal dissection. Blood flow within the false lumen is generally slower, leading to turbulence, and appears as heterogeneous signals on MRI, commonly showing hematoma formation (20, 21, 30, 31).

Intracranial magnetic resonance angiography (MRA) is also one of the commonly used tools for diagnosing MCA dissection. MRA is a non-invasive examination suitable for long-term follow-up. MRA shows that the affected segment of the MCA is irregular and unclear, with a finer diameter at both proximal and distal ends, indicating stenosis. Similarly, in the stenotic area, sagittal T2-weighted and T1-weighted pre-contrast vascular wall magnetic resonance imaging (VWI) reveals eccentric high signal and eccentric thickening of the vessel wall. The MRA showed multiple round and oval high density shadows within the wall of the MCA, resembling a string of beads, known as the bead sign (21, 32, 33).

Magnetic resonance imaging (MRI) has become the modality of choice for evaluating cerebral artery dissections due to its unique combination of advantages, including absence of ionizing radiation, multi-parametric imaging capabilities (T1/T2/TOF/contrast-enhanced sequences), and superior vessel wall visualization with 0.4 mm resolution, allowing for detailed assessment of intramural hematomas and intimal flaps. However, its clinical utility is somewhat limited in acute settings by relatively long acquisition times (typically 20–45 min) and poor sensitivity for detecting vascular calcifications, which may necessitate complementary CT angiography in certain cases. These characteristics make MRI particularly valuable for subacute and chronic phase monitoring, as well as for serial follow-up examinations where repeated imaging is required, though its limitations in acute stroke evaluation highlight the importance of a multimodal imaging approach tailored to specific clinical scenarios and time-sensitive diagnostic needs (21, 27, 30).

#### Computed tomography

Computed tomography (CT) and CT angiography (CTA) serve as essential diagnostic tools for intracranial artery dissection, offering distinct advantages in acute clinical settings. Non-contrast CT remains the first-line imaging modality for initial evaluation of acute neurological deficits, primarily to exclude hemorrhagic complications (particularly subarachnoid hemorrhage visible as hyperdense sulcal effacement) and to assess patients with contraindications to contrast administration. Employing CTA for direct dissection characterization, key findings include: (1) intimal flaps appearing as linear hypodensities traversing the lumen (sensitivity 65–75%), (2) intramural hematomas demonstrating crescentic hyperattenuation (50–70 Hounsfield units), and (3) less frequently visualized double-lumen signs compared to DSA (detection rate 30–45%). Indirect CTA manifestations encompass luminal abnormalities (abrupt caliber changes or tapered occlusions) and secondary ischemic sequelae—territorial hypodensity indicating infarction or focal sulcal hyperdensity signaling SAH. The modality’s rapid acquisition (90 s) and ubiquitous availability make it indispensable for emergency decision-making, though its inferior soft-tissue contrast compared to MRI limits detailed wall characterization (27, 28).

#### Doppler

Doppler ultrasonography demonstrates approximately 90% sensitivity in detecting carotid artery dissections, utilizing multiple techniques including continuous-wave Doppler velocimetry, standard duplex imaging, and color Doppler flow mapping. The most consistent hemodynamic finding is a high-resistance flow pattern in distal arterial segments, which, when combined with typical clinical presentation and absence of atherosclerotic changes, strongly suggests dissection. While pathognomonic features like intramural hematoma, double lumen, or intimal flap are infrequently visualized, transcranial Doppler may aid in intracranial dissection assessment. This modality offers advantages of non-invasiveness, cost-effectiveness, and widespread availability, making it suitable for serial monitoring. However, technical limitations include difficulty evaluating distal internal carotid segments, limited emboli detection capability, and reduced sensitivity for low-grade stenosis dissections. Therefore, transcranial Doppler ultrasound provides another way of diagnosing MCA dissection. In isolated MCA dissection, transcranial Doppler ultrasound found that the blood flow velocity of the ipsilateral middle cerebral artery was significantly increased compared with supine position, which may aggravate headache (20, 28).

Diagnostic criteria (met at least one of the following):

1. DSA: Identify double lumen sign/endocardial flap.
2. HR-MRI: intracerebral hematoma (high signal on T1WI) + vascular stenosis.
3. CTA + highly suspected clinical findings: beaded stenosis + sudden neurological deficit + no evidence of atherosclerosis.

**Figure 3:**
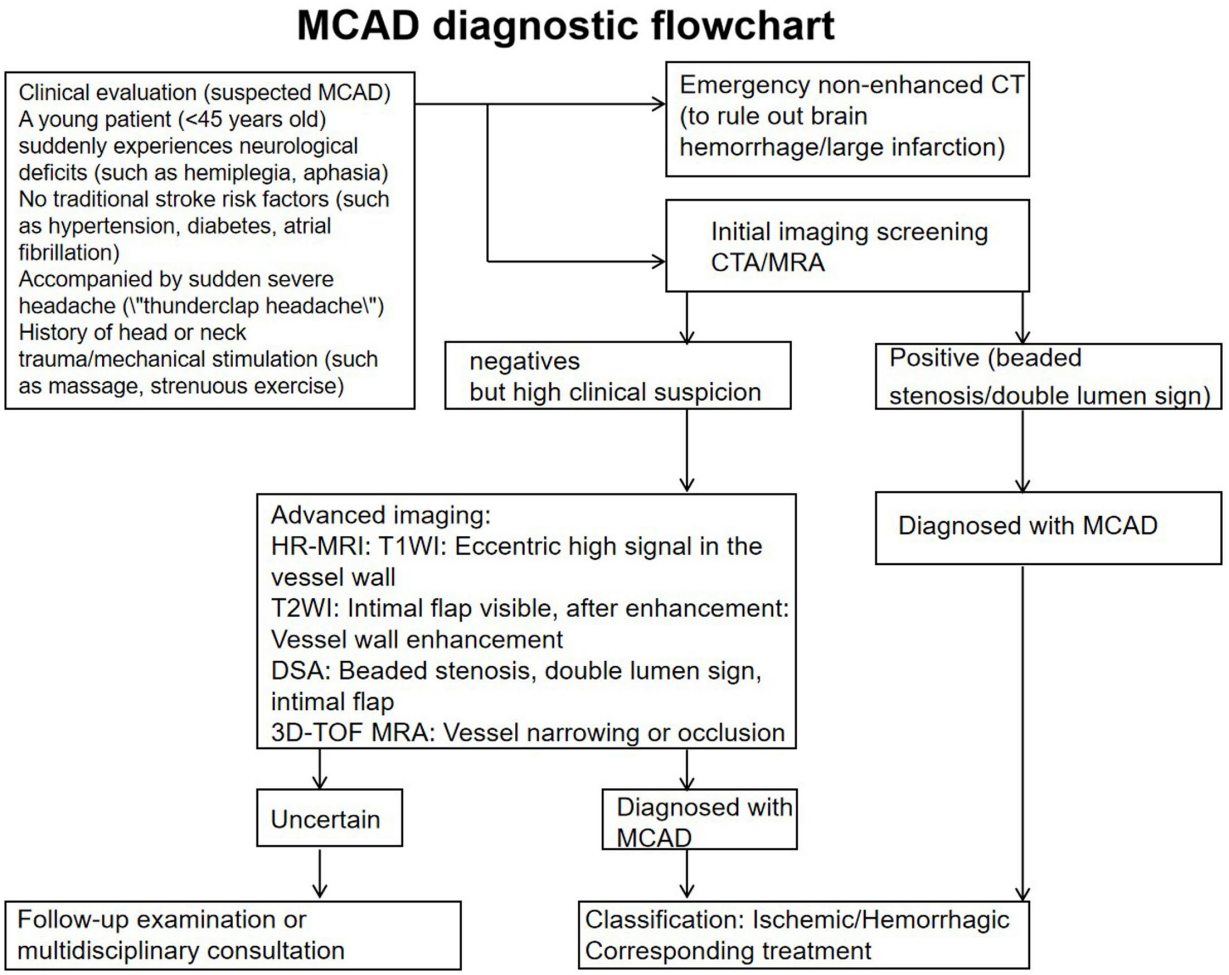
Diagnostic flowchart for MCAD.

Intracranial artery dissection risk scores ≤5 and d-dimer negative (threshold 500 ng/mL) can accurately and efficiently rule out MCAD. sST2 levels can serve as an exclusion marker, potentially even better than d-dimer to some extent. Elevated C-reactive protein and granulocyte-macrophage colony-stimulating factor levels. Additionally, experimental evidence indicates the contribution of granulocyte colony-stimulating factor and interleukin-17 (Figure 3) (34).

#### Treatment

The management of middle cerebral artery dissection (MCAD) remains clinically challenging due to the lack of standardized protocols, requiring individualized treatment strategies based on lesion characteristics and clinical manifestations. For ischemic presentations (accounting for 80–90% of cases), conservative medical management with antiplatelet agents (aspirin/clopidogrel) or anticoagulation (heparin/warfarin/DOACs) forms the mainstay of therapy, combined with strict blood pressure control (target SBP < 140 mmHg). DOACs (Direct Oral Anticoagulants, Direct Oral Anticoagulants), also often referred to in clinical practice as NOACs (Non-Vitamin K Antagonist Oral Anticoagulants, Non-Vitamin K Antagonist Oral Anticoagulants), are a new class of anticoagulants. The main drugs in this class include: rivaroxaban, apixaban, edoxaban, and dabigatranate. In contrast, hemorrhagic complications (10–20% of cases) or large pseudoaneurysms (>7 mm) typically necessitate surgical intervention, including microsurgical clipping for ruptured dissections or endovascular approaches (stenting/coiling) for accessible lesions (35).

Conservative management.

The conservative management of middle cerebral artery dissection (MCAD) encompasses a spectrum of non-surgical pharmacological strategies tailored to clinical presentation and temporal factors. For patients with mild or stable neurological deficits and absence of hemorrhage, initial medical therapy is preferred, employing either antiplatelet monotherapy (aspirin 100 mg/day + clopidogrel 75 mg/day) or anticoagulation (low molecular weight heparin bridging to warfarin, target INR 2–3). In cases of prolonged ischemia (4.5 h from symptom onset), intravenous thrombolysis is contraindicated; instead, dual antiplatelet therapy (DAPT) with parenteral anticoagulation (enoxaparin 1 mg/kg BID) is initiated, followed by gradual transition to monotherapy based on serial imaging (typically MRI at 72 h) and clinical response. For subacute presentations (6 h) with partial vessel patency despite large infarcts, protocol-based intravenous alteplase (0.9 mg/kg) may be considered, supplemented with adjunctive antiplatelet/anticoagulant therapy if no hemorrhagic transformation occurs (35–38).

The safety of thrombolysis has not been confirmed. While thrombolysis can reverse the ischemic pathological process of MCAD, it may also lead to hematoma expansion, pseudoaneurysm formation, and subarachnoid hemorrhage, so its use should be cautious. It is worth noting that compared to extracranial arterial dissection, antiplatelet therapy is safer than anticoagulation therapy, a consensus that has been reached., heparin, warfarin, DOACs According to the AHA/ASA guidelines, anticoagulation (e.g., heparin, warfarin, DOACs) is not a first-line option for acute ischemic stroke (AIS), and the routine use of anticoagulation (e.g., heparin, warfarin, DOACs, heparin) is not recommended as an alternative to intravenous thrombolysis or endovascular therapy in the acute phase (Class III recommendation, no clear benefit). The main reason for this is that anticoagulants do not rapidly open occluded large vessels and may instead increase the risk of intracranial or systemic hemorrhage (37, 39).

The novel intravenous P2Y12 receptor antagonist Canrevieza represents a paradigm shift in the antithrombotic management of middle cerebral artery dissection (MCAD), particularly for high-bleeding-risk patients. This next-generation antiplatelet agent achieves 80% P2Y12 receptor blockade within 5 min of intravenous administration while offering uniquely rapid reversibility, with platelet function recovering to >50% baseline within 60 min after infusion cessation—a critical safety feature for neurovascular interventions. Its clinical utility is further enhanced by a validated transition protocol to oral ticagrelor (180 mg loading dose administered 30 min prior to IV discontinuation), which maintains >70% receptor coverage during bridging compared to conventional agents. Ongoing clinical trials (MARVEL-ICH, PRINCE-II) are further evaluating its efficacy and safety profiles in neurovascular pathologies, with preliminary data suggesting reduced hemorrhagic complications without compromising anti-ischemic efficacy compared to traditional heparin bridging regimens (40).

#### Surgical management

Surgical intervention represents a definitive yet invasive approach for managing MCA dissection, encompassing a range of techniques including microsurgical clipping, wrapping/resection, trapping (with or without revascularization), and endovascular therapy (e.g., stenting, flow diversion). While current strategies are largely extrapolated from extracranial dissection protocols, the primary objectives remain: (1) restoration of physiological blood flow, (2) preservation of cerebral perfusion, (3) prevention of thromboembolic events, and (4) mitigation of hemorrhagic risk (35, 39, 40).

Surgical intervention for middle cerebral artery (MCA) dissection is primarily indicated in four clinical scenarios: (1) refractory cases progressive or recurrent ischemic symptoms despite optimal medical therapy (antiplatelets/anticoagulants), (2) complete MCA occlusion presenting with acute neurological deficits (NIHSS ≥6), (3) rapidly expanding dissecting aneurysms (7 mm) or vessel occlusion, and (4) hemorrhagic presentations including subarachnoid hemorrhage or intraparenchymal hematoma from rupture (39, 41).

Clamping is the first-line treatment for cystic MCA aneurysms; however, this procedure is extremely difficult and carries significant risks (42).

Endovascular management of dissection-induced vascular stenosis employs balloon angioplasty with possible stent placement to achieve luminal reconstruction. Under fluoroscopic guidance, a balloon catheter is advanced over a guidewire to dilate the stenotic segment, followed by stent deployment if suboptimal expansion persists, with the dual objectives of false lumen obliteration and true lumen recanalization to restore physiological perfusion. This approach carries two principal peri-procedural risks: (1) iatrogenic dissection propagation resulting from inadvertent guidewire passage into the false lumen, which may extend the primary intimal tear, and (2) reperfusion injury syndrome characterized by disruption of cerebrovascular autoregulation and hemorrhagic transformation following revascularization. The latter complication reflects abrupt restoration of pressure gradients across previously hypoperfused vascular beds with impaired endothelial integrity, while the former underscores the technical imperative for true lumen navigation verified by intravascular ultrasound or high-resolution cone-beam CT during intervention (43).

For acute middle cerebral artery (MCA) dissection with complete occlusion, semi-shadow aspiration thrombectomy has emerged as the preferred first-line endovascular intervention, offering distinct advantages in mitigating procedural risks. Passage of microcatheters and microguidewires through MCA occlusions associated with MCA entrapment should be done with great care because the site of entry of the true or false lumen is not known. If the microcatheter enters the false lumen, it may lead to extensive stretching of the false lumen and rupture of the intracranial artery. We can use a method of embolization that does not pass through the microcatheter-semi-shadow aspiration thrombectomy. Upon confirming occlusion, the semi-shadow catheter is precisely positioned at the thrombus interface for controlled aspiration, achieving TICI 2b/3 recanalization in 70–80% of cases while minimizing reperfusion injury risks. Post-procedural angiography provides essential evaluation of true lumen restoration, residual false lumen status, and collateral circulation integrity, with studies demonstrating significantly improved functional outcomes (mRS ≤ 2 at 90 days) when performed within the 6-h therapeutic window. The procedure’s safety profile stems from reduced mechanical wall stress compared to stent-retriever thrombectomy, though optimal post-interventional antithrombotic management and long-term vessel remodeling outcomes require further investigation (22, 44).

Patients with acute hemorrhagic middle cerebral artery (MCA) dissection demonstrate prohibitively high rebleeding rates and mortality with conservative management, mandating urgent surgical or endovascular intervention. The contemporary therapeutic paradigm offers two principal approaches: (1) definitive occlusion via surgical trapping or endovascular parent artery embolization (effective in >85% of cases but carrying 10–15% infarction risk in bypass-dependent territories), and (2) reconstructive techniques employing stent-assisted coiling (achieving complete occlusion in 60–70% of cases at 6-month follow-up). Endovascular procedures, performed under general anesthesia via femoral access with systemic heparinization (ACT 250–300 s), utilize 3D-DSA roadmapping for precise microcatheter navigation to the dissection site, followed by tailored coil embolization. While stent deployment improves anatomical results (TICI 2b/3 in 70–75%), the requisite dual antiplatelet therapy (DAPT) elevates early hemorrhagic complication rates to 15–25% during the vulnerable SAH period. Post-interventional imaging critically informs further management, with residual lesions >3 mm often necessitating delayed surgical revision. This risk-stratified approach balances the imperative for definitive hemorrhage prevention against the periprocedural challenges of maintaining vessel integrity and cerebral perfusion (38, 42, 45, 46).

**Figure 4:**
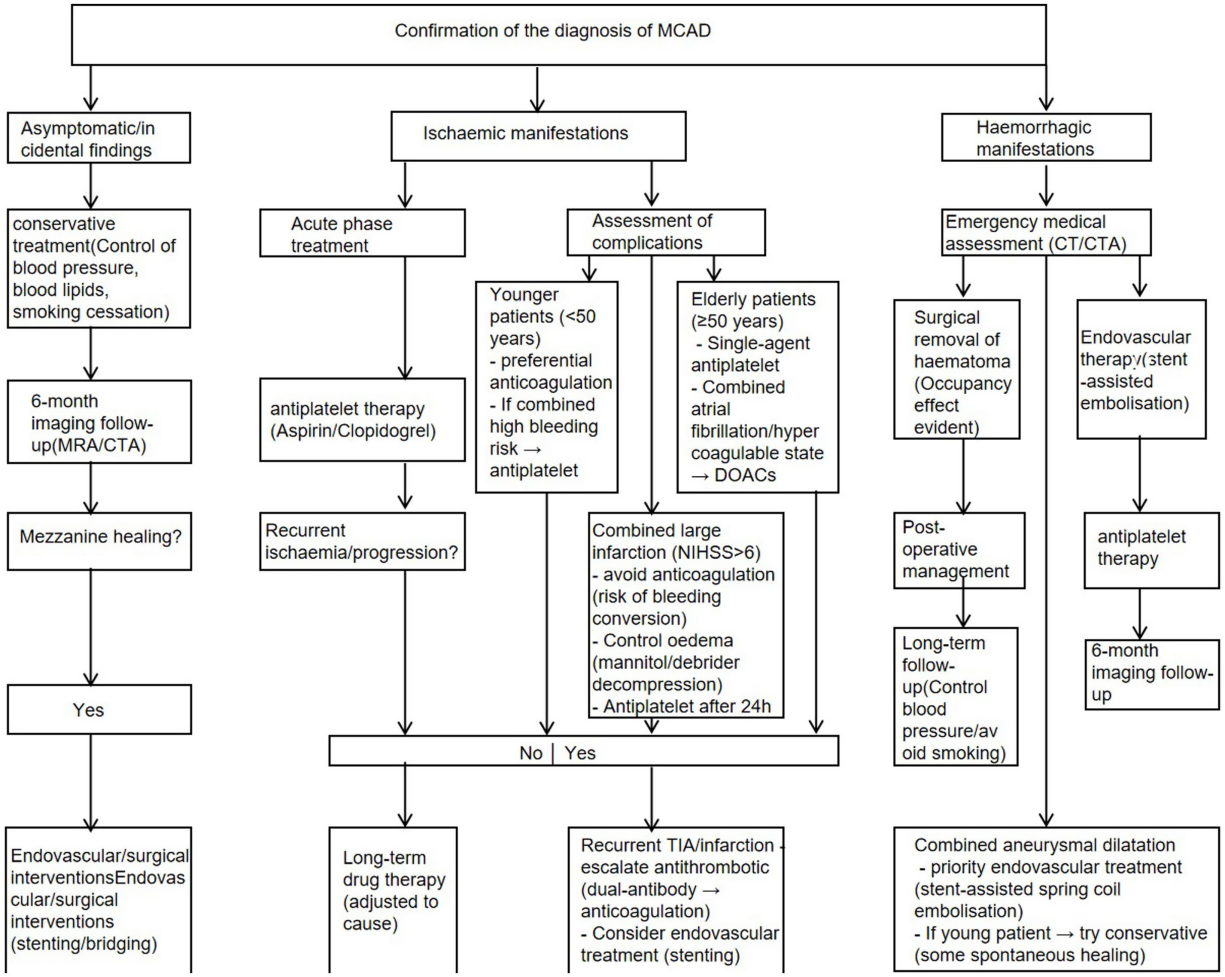
Therapeutic flowchart for MCAD.

The flow chart of the treatment is shown at Figure 4.

## Discussion

Middle cerebral artery dissection arises from the vessel’s inherent anatomical vulnerabilities, including its thin medial layer (0.1–0.3 mm), absent external elastic lamina, and hemodynamic stress concentration at the M1-M2 junction. Major risk factors comprise connective tissue disorders (OR 6.2), cervical trauma (OR 4.5), hypertension (OR 1.8), and recent infections (OR 2.1), which collectively trigger a pathogenetic cascade initiating with internal elastic lamina rupture and progressing to intramural hematoma formation. Clinically, MCAD manifests as either ischemic (88% of cases) or hemorrhagic (12%) syndromes, with the former presenting as sudden-onset contralateral hemiparesis (NIHSS 8–15) and cortical deficits, while the latter exhibits thunderclap headache with rapid neurological decline (GCS ≤ 12 in 65%).

Digital subtraction angiography (DSA) remains the gold standard for MCAD detection, offering superior temporal resolution (7–15 frames/s) to identify pathognomonic signs such as the double lumen (50–60% sensitivity) and intimal flap (65–75% sensitivity), though its invasive nature limits routine use. CT angiography (CTA) provides rapid acquisition (90 s) and excellent spatial resolution (0.5–0.6 mm isotropic voxels), reliably demonstrating indirect signs like tapered stenosis (85–92% sensitivity) and intramural hematoma (50–70 HU density), but suffers from lower specificity (70–80%) due to limited wall visualization. High-resolution MRI (HR-MRI) excels in vessel wall characterization (0.4 mm resolution), clearly depicting intramural hematoma (T1 crescentic hyperintensity) and differentiating dissection from atherosclerosis (eccentric vs. concentric wall thickening), albeit with longer scan times (20–45 min) and contraindications in acute emergencies. While DSA is indispensable for endovascular planning, CTA serves as the first-line tool in acute settings, and HR-MRI offers unparalleled subacute/chronic phase assessment. The optimal approach integrates these modalities based on clinical urgency, with DSA confirming equivocal cases, CTA ruling out hemorrhage, and HR-MRI evaluating wall pathology.

For ischemic strokes caused by vascular stenosis due to MCAD, drug therapy can be used, with antiplatelet drugs being the first choice, such as aspirin and clopidogrel. Anticoagulation therapy is controversial. Intravenous thrombolysis is considered safe in patients with carotid artery dissection with ischemic stroke, and is therefore considered safe in patients with intracranial dissection, although the supporting data are limited to case reports. The American Heart Association (AHA)/American Stroke Association (ASA) guidelines recommend that the standard time window for intravenous thrombolysis (alteplase) in patients with ischemic stroke is 3 h after onset of the stroke (Class I recommendation), which may be extended to 4.5 h in some carefully screened patients (Class I recommendation). Exclusion criteria for an extended time window include age >80 years, taking oral anticoagulants, NIHSS score >25, history of diabetes mellitus in combination with a previous stroke, or large infarcts on imaging. Indications were confirmed ischemic stroke with disabling neurological deficit (NIHSS ≥1) and imaging (CT/MRI) to exclude intracranial hemorrhage. Contraindications included recent intracranial hemorrhage or surgery, active internal bleeding, coagulation disorders, poor blood pressure control (185/110 mmHg), and abnormal blood glucose (<50 mg/dL or >400 mg/dL). Clinical decision-making needs to incorporate individualized assessment to ensure the safety and efficacy of thrombolytic therapy.(53, 54)3Intravenous thrombolytic therapy is primarily administered within 4.5 h of the onset of symptoms. Intravenous thrombolytic therapy may be considered in patients with large vessel occlusions, while recent studies have shown that there are no contraindications to endovascular recanalization in this group of patients. Thus endovascular thrombectomy has achieved the same importance as intravenous thrombolysis (47). Surgical interventions such as aneurysm repair, external ventricular drainage for hydrocephalus, or decompression for herniation may be required when a patient develops SAH. Surgery or endovascular treatment should be considered for those patients who continue to experience ischemic symptoms even after adequate anticoagulation. Surgical treatment usually involves ligation of the carotid or vertebral arteries in combination with *in situ* or extracranial to intracranial bypass surgery (48).

Drug therapy can also be used preoperatively, intraoperatively, and postoperatively to support surgery and improve outcomes. Perioperative drug therapy for middle cerebral artery dissection needs to be adjusted according to the type of procedure and patient individualization. For preoperative stenting, dual antiplatelet therapy (aspirin 100 mg + clopidogrel 75 mg) should be started 5–7 days in advance, and a loading dose (300 mg of each) can be given in case of emergency surgery; long-term anticoagulants should be bridged, and patients with warfarin should be bridged with low molecular heparin, and patients with DOACs should stop their medication 24–48 h prior to the surgery. Intraoperative endovascular manipulation requires heparinization (70–100 IU/kg) and maintenance of ACT for 250–300 s, along with prevention of vasospasm with nimodipine. Postoperative stent patients need to continue dual-antibody for 3–6 months and then change to single-antibody for long-term maintenance, and those with high thrombotic risk can extend dual-antibody to 12 months; those who need anticoagulation for comorbidities, such as atrial fibrillation, can be coadministered with warfarin (INR2-3) or DOACs, but need to shorten the duration of dual-antibody. Blood pressure is controlled throughout, with SBP < 140 mmHg maintained preoperatively and postoperatively with nicardipine or uradil; statins (e.g., atorvastatin 40 mg) need to be used long-term. Bleeding complications require immediate neutralization and anticoagulation, blood pressure control, and surgical removal of hematoma if necessary. Special populations such as renal insufficiency need to adjust the anticoagulant dosage, and antiplatelet and anticoagulant dosages are adjusted according to body weight in pediatric patients. Postoperatively, blood routine, liver and kidney function and platelet function need to be closely monitored, and regular imaging follow-up.

For the acute phase of MCAD hemorrhagic stroke, surgical and interventional treatments are recommended. Hemorrhagic cases are often caused by subarachnoid hemorrhage due to the rupture of a dissecting aneurysm, for which endovascular treatment is preferred, using coils to occlude the ruptured vessel. Iatrogenic dissection should be considered; during the experience of reconstructing occluded or narrowed MCA arteries, wall damage may occur during wire passage, catheter transport, PTA, stent placement, or retrieval of devices used for distal serial occlusion, potentially leading to vascular dissection. Therefore, the management of iatrogenic dissection should be given attention. For dissections caused by stent placement or balloon dilation, a more flexible stent can be inserted for treatment. For dissections resulting from mechanical thrombectomy, the degree of blood flow restriction must be immediately assessed, which can be done through intraoperative DSA or high-resolution MRI. If the dissection is mild, conservative treatment with intraoperative heparinization combined with postoperative antiplatelet therapy can be used. If severe dissection occurs, emergency stent or covered stent implantation to restore blood flow is required, and anticoagulation therapy may be necessary if needed. The case of failure of conventional coil-assisted stent encircling treatment for laminar aneurysm, a neurography Y-type stent can be used as salvage treatment. This catheter has a small profile and can be deployed through a low-profile catheter, greatly reducing intraoperative complications (49).

## Conclusion

Aneurysm of the middle cerebral artery is a critical condition that can lead to both acute ischemic stroke and hemorrhagic disease, making it a disabling illness. It requires timely identification and standardized diagnosis according to established procedures. Its imaging features are diverse, including biconvexity, intimal flap, beaded sign, direct signs of intraluminal hematoma, and other indirect signs. Treatment options should be selected based on different imaging characteristics.
